# Continuous 24-h Photoplethysmogram Monitoring Enables Detection of Atrial Fibrillation

**DOI:** 10.3389/fphys.2021.778775

**Published:** 2022-01-04

**Authors:** Eemu-Samuli Väliaho, Jukka A. Lipponen, Pekka Kuoppa, Tero J. Martikainen, Helena Jäntti, Tuomas T. Rissanen, Maaret Castrén, Jari Halonen, Mika P. Tarvainen, Tiina M. Laitinen, Tomi P. Laitinen, Onni E. Santala, Olli Rantula, Noora S. Naukkarinen, Juha E. K. Hartikainen

**Affiliations:** ^1^School of Medicine, Faculty of Health Sciences, University of Eastern Finland, Kuopio, Finland; ^2^Doctoral School, Faculty of Health Sciences, University of Eastern Finland, Kuopio, Finland; ^3^Department of Applied Physics, Faculty of Science and Forestry, University of Eastern Finland, Kuopio, Finland; ^4^Department of Emergency Care, Kuopio University Hospital, Kuopio, Finland; ^5^Center for Prehospital Emergency Care, Kuopio University Hospital, Kuopio, Finland; ^6^Heart Center, North Karelia Central Hospital, Joensuu, Finland; ^7^Department of Emergency Medicine, University of Helsinki, Helsinki, Finland; ^8^Department of Emergency Medicine and Services, Helsinki University Hospital, Helsinki, Finland; ^9^Heart Center, Kuopio University Hospital, Kuopio, Finland; ^10^Department of Clinical Physiology and Nuclear Medicine, Kuopio University Hospital, Kuopio, Finland; ^11^Imaging Center, Kuopio University Hospital, Kuopio, Finland

**Keywords:** atrial fibrillation, photoplethysmography, photoplethysmogram, quality, algorithms, monitoring, screening, signal quality analysis

## Abstract

**Aim:** Atrial fibrillation (AF) detection is challenging because it is often asymptomatic and paroxysmal. We evaluated continuous photoplethysmogram (PPG) for signal quality and detection of AF.

**Methods:** PPGs were recorded using a wrist-band device in 173 patients (76 AF, 97 sinus rhythm, SR) for 24 h. Simultaneously recorded 3-lead ambulatory ECG served as control. The recordings were split into 10-, 20-, 30-, and 60-min time-frames. The sensitivity, specificity, and F1-score of AF detection were evaluated for each time-frame. AF alarms were generated to simulate continuous AF monitoring. Sensitivities, specificities, and positive predictive values (PPVs) of the alarms were evaluated. User experiences of PPG and ECG recordings were assessed. The study was registered in the Clinical Trials database (NCT03507335).

**Results:** The quality of PPG signal was better during night-time than in daytime (67.3 ± 22.4% vs. 30.5 ± 19.4%, *p* < 0.001). The 30-min time-frame yielded the highest F1-score (0.9536), identifying AF correctly in 72/76 AF patients (sensitivity 94.7%), only 3/97 SR patients receiving a false AF diagnosis (specificity 96.9%). The sensitivity and PPV of the simulated AF alarms were 78.2 and 97.2% at night, and 49.3 and 97.0% during the daytime. 82% of patients were willing to use the device at home.

**Conclusion:** PPG wrist-band provided reliable AF identification both during daytime and night-time. The PPG data’s quality was better at night. The positive user experience suggests that wearable PPG devices could be feasible for continuous rhythm monitoring.

## Introduction

Atrial fibrillation (AF) is the most common sustained cardiac arrhythmia worldwide and an independent risk for stroke (Hariri et al., 2016; Morillo et al., 2017; Hindricks et al., 2021). The prevalence of AF is increasing due to the aging population, an epidemic is predicted to occur in the next few years resulting in a significant burden on health care systems (Morillo et al., 2017). Early detection of AF and timely initiated anticoagulation reduces AF related mortality and morbidity (Morillo et al., 2017). Unfortunately, the detection of AF is often challenging, up to 30% of AF patients are asymptomatic, (Hariri et al., 2016), and 25–60% of all AFs are paroxysmal (Hariri et al., 2016). Thus, despite a comprehensive clinical examination, almost 30% of all strokes remain cryptogenic and occult paroxysmal AF is one of the culprits (Hariri et al., 2016).

Ambulatory 24-h ECG monitoring is often used for AF screening, such as in patients with cryptogenic stroke, with palpitations or after cardiac surgery. Nonetheless, 24-h recording time is suboptimal when searching for paroxysmal AF although the diagnostic yield increases with prolonged duration and increased number of screenings (Hariri et al., 2016; Diederichsen et al., 2020). It has been claimed that prolonged monitoring improves the possibility of detection of AF and reduces health resource utilization and costs, especially in patients with heart failure and post-stroke (Morillo et al., 2017). Recently, the number of subjects needed to screen was found to be 21 for screening of AF with a 2-week ECG patch capable of continuous rhythm monitoring amongst patients aged ≥75 years with no previously diagnosed AF (Gladstone et al., 2021). The increased mortality, morbidity and economic burden caused by AF justifies screening of the elderly population (Hindricks et al., 2021). However, long-term, ambulatory ECG involves burdensome wires and adhesive electrodes causing skin irritation (Hariri et al., 2016).

Mobile health technologies are being increasingly developed for the detection of paroxysmal and silent AF, but many of the currently available consumer-grade automated arrhythmia detection technologies can only provide intermittent single rhythm analyses. Photoplethysmography has been proposed as one of the most favorable and cost-effective methods (Hindricks et al., 2021). Wearable PPG devices, such as wrist-band devices, could provide a comfortable, continuous long-term monitoring without electric wires or adhesive patches when searching for an asymptomatic and paroxysmal AF. An AF alarm generated by PPG-based rhythm analysis would be followed by a timely ECG recording for the final diagnosis of AF. Here, we assessed whether a PPG wrist-band device could be used for the reliable detection of AF with ambulatory ECG serving as the golden standard.

## Materials and Methods

### Study Population

**Figure 1:**
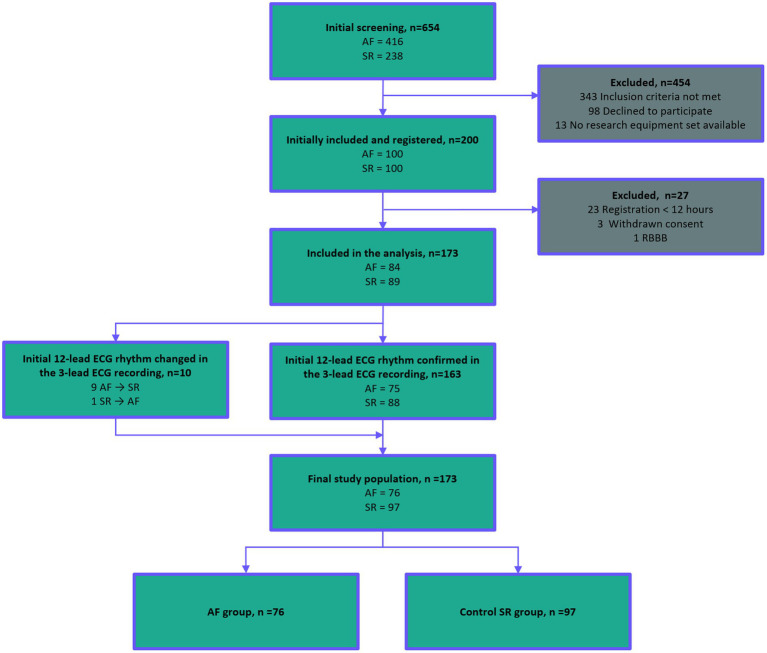
Standards for Reporting Diagnostic Accuracy Studies (STARD) flow diagram of the study patient flow. A total of 654 patients were screened in Kuopio University Hospital. 200 patients were initially recruited, and 173 patients were accepted in the final study population. Abbreviations: AF, atrial fibrillation; ECG, electrocardiogram; SR, sinus rhythm; PPG, photoplethysmography; and RBBB, right bundle branch block. STARD 2015 checklist and information about STARD 2015 is presented in Supplementary Material.

The study was conducted in Kuopio University Hospital. Altogether 654 patients were screened for the study in emergency care department and acute wards between April 2018 and December 2019. The inclusion criteria were AF or SR diagnosed in the 12-lead resting ECG upon arrival, age ≥ 18 years and estimated in-hospital treatment ≥24 h. The exclusion criteria were body mass index (BMI) ≥ 35 kg/m^2^, permanent cardiac pacemaker, left or right bundle branch block, or a medical condition requiring immediate treatment. Initially, 200 patients were recruited in the study but 23 patients with 3-lead ambulatory ECG or PPG recording less than 12 h, three who withdrew their consent and one with RBBB were excluded. Thus, the final study population consisted of 173 patients; 76 patients were assigned to the AF group and 97 patients to the control SR group (Figure 1). Graphical abstract of the study is presented in Supplementary Material.

### Data Acquisition

The 12-lead resting ECG recorded upon hospital admission and interpreted by the treating physician was used for screening.

**Figure 2:**
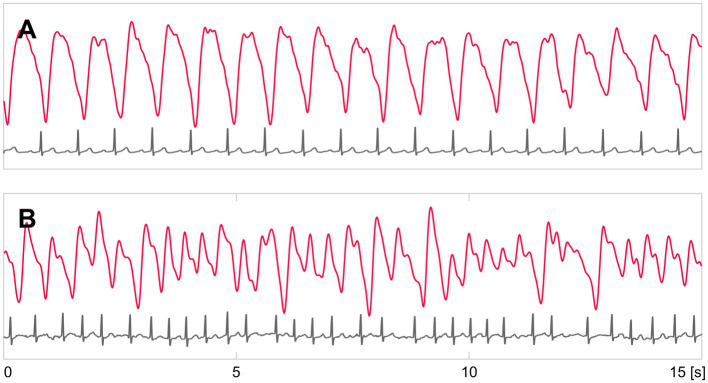
Example recordings of PPG (red) and ECG (gray) signals. **(A)** shows recordings of a patient in SR, and **(B)** in AF.

The 3-lead ambulatory ECG and PPG were recorded simultaneously for 24 h. The ECG was recorded using a Holter ECG device (Faros 360, Bittium, Oulu, Finland). The PPG was recorded with an Empatica E4 wrist-band (Empatica Inc., Cambridge, United States) from the non-dominant hand wrist. Examples of PPG and ECG recordings are presented in Figure 2. The acceleration data were obtained with the acceleration sensor of the Empatica E4 wrist-band. The patient was lying supine for at least 2 min before and 5 min after the start of the recordings. Subsequently, the patient could move freely during the study recordings; these were discontinued after 24 h or earlier if the patient was discharged from the hospital. Clinical data were collected by interviewing the patient and from medical records. Possible symptoms and discomfort during the ECG and PPG recording were collected with a survey.

### 3-Lead Ambulatory ECG Analysis

The 3-lead ambulatory ECG signal was recorded with 250 Hz sampling frequency. Leads I, II, and V3 were recorded. The ambulatory ECG recording was first automatically analyzed with the Medilog^®^ Darwin Professional 2 software version 2.8.1 (Schiller Global AG, Baar, Switzerland). Subsequently, the rhythm was evaluated independently by four investigators blinded to the initial 12-lead resting ECG. The rhythm was classified as AF or SR. QRS-complexes were automatically detected by the Darwin software and the classification into sinoatrial beats and extrasystoles was revised and corrected manually. The 3-lead ambulatory ECG served as the golden standard for the final rhythm analysis.

### Pre-processing of the PPG

The PPG signal was recorded with a 64 Hz sampling frequency. The PPG data were pre-processed and analyzed using MATLAB^®^ software version R2017b. The raw PPG signal was first interpolated at 128 Hz with cubic spline interpolation to increase the time resolution of the pulse detection. PPG pulse detection was based on a method validated in our previous study (Väliaho et al., 2019). Baseline drift and high frequency noise were removed with a 0.1–7 Hz bandpass filter.

### PPG Quality Analysis

A PPG quality algorithm was validated with 50 patients of the study population. Validation results are presented in Supplementary Material. PPG quality algorithm achieved 92.7% PPV for separating high-quality 1-min samples from low-quality samples.

PPG recordings of each patient were split into consecutive 1-min segments. A quality algorithm interpreted PPG segments of acceptable quality if the acceleration sensor indicated none or little hand-movement (root mean square of acceleration < 1 m/s^2^), and if the PPG signal was stationary. The quality of the PPG data obtained in patients with AF and SR was compared. Furthermore, we evaluated PPG signal quality during daytime (7:00 AM–7:00 PM) and at night (7.00 PM–7:00 AM).

### Detection of AF and Simulated Alarms

The AF detection algorithm validated previously (Väliaho et al., 2021) was used for the evaluation of continuous AF monitoring. The diagnostic performance of four different AF detector time-frames of 10, 20, 30, and 60 min were evaluated from the 24-h recordings. Sensitivities and specificities of all time-frames were evaluated for detection of AF as time-based and patient-based. The time-based sensitivities and specificities were calculated from interpretable PPG data and based on AF (and SR) episodes. The patient-based sensitivities and specificities for AF detection were calculated with correctly and incorrectly diagnosed cases.

AF alarms were generated for each patient, respectively, using the time-frames of 10, 20, 30, and 60 min. Each time-frame was “slided” through the 24-h PPG minute by minute for each patient. An AF alarm was generated when the conditions were met if >40% of its 1-min samples were of sufficient quality by the quality algorithm and >85% of these sufficient quality samples were classified as AF by the AF detection algorithm. These AF alarms were evaluated for each hour to simulate the performance of a continuous rhythm monitoring method for AF screening. AF alarm mean sensitivity and PPV were calculated as patient-based for each hour. In addition, for the time-frame with the highest F1-score, mean values were calculated for both day (7:00 AM–7:00 PM) and night (7:00 PM–7:00 AM) time, and for the whole day (7:00 AM–7:00 AM).

### User Experience

User experience of the devices was assessed with a patient survey. Participants were asked how comfortable the devices were on a semantic differential scale 1–5 (1 = comfortable, 2 = reasonably comfortable, 3 = neutral, 4 = slightly uncomfortable, and 5 = uncomfortable). Patients were also asked if the device interfered with their daily activities, such as moving, eating, sleeping, and going to the toilet, and would they be willing to use the device voluntarily at home for rhythm monitoring.

### Statistical Analysis

In the power calculation, it was estimated that 200 observations with an assumed sensitivity of 95% and with a 3% margin error would be sufficient. We used IBM SPSS statistics software version 25 in the statistical analysis. The differences between the AF and the SR groups were tested with chi-squared or Fischer’s exact tests and *T*-tests. The comparison between the PPG quality proportions during the day and night within groups, and differences between the comfort of use between the wrist-band and Holter device were tested with paired samples T-tests. Group statistics were expressed as means and standard deviations or numbers and percentages with differences and 95% confidence intervals. Two-sided values of *p* ≤ 0.05 were considered statistically significant.

F1-score analyses were executed for 10-, 20-, 30-, and 60-min time-frames for detection of AF. In addition, sensitivities and specificities were evaluated. The F1-score is the harmonic mean of precision and recall. Sensitivities and PPVs were evaluated for AF alarms for each hour.

## Results

### Clinical Characteristics of the Patients

The study population consisted of 173 patients; 76 patients were assigned to the AF group and 97 patients to the SR group (Figure 1). Eleven (14.5%) patients in the AF group presented with paroxysmal AF. One of the paroxysmal AF patients had 34 AF episodes, one had 3, one had 2, and the rest eight of the patients had only 1 AF episode. Median AF paroxysm duration was 6 min 45 s, minimum 30 s and maximum duration 20 h.

AF patients were older and had more often a history of AF, hypertension, congestive heart failure, and cardiac surgery, and they were more often receiving treatment with anticoagulants, beta-blockers, and/or digoxin. AF patients reported respiratory distress and heart palpitations prior to hospital admission more often than the SR patients (Table 1).

**Table 1:** Patient demographics.

		AF group (*n* = 76)	SR group (*n* = 97)	Significance (2-sided)	Mean difference and (95% CI of the difference)
Characteristics	Age, years	77.1 ± 9.7	67.3 ± 15.8	<0.001	9.75 (5.89 – 13.60)^*^
	BMI, kg/m^2^	27.0 ± 4.6	26.1 ± 4.2	0.180	0.90 (−0.42 – 2.23)^*^
	Sex, male	41 (53.9)	43 (44.3)	0.209	−0.10 (−0.24 – 0.05)
Recording	Heart rate (ECG), 1/min	78.0 ± 18.8	66.5 ± 11.8	<0.001	11.51 (6.63 – 16.39)^*^
	Duration, h	22.0 ± 3.0	21.8 ± 3.4	0.705	0.19 (−0.79 – 1.16)^*^
Medical history	Earlier AF diagnosis	61 (80.3)	18 (18.6)	<0.001	−61.71 (−71.54 – −48.10)
	Hypertension	57 (75.0)	55 (56.7)	0.012	−18.30 (−31.29 – −3.99)
	Congestive heart failure	37 (48.7)	9 (9.3)	<0.001	−39.41 (−51.25 – −26.22)
	Coronary artery disease	27 (35.5)	25 (25.8)	0.165	−9.75 (−23.34 – 3.92)
	Diabetes	20 (26.3)	20 (20.6)	0.378	−5.70 (−18.54 − 6.81)
	Prior cardiac surgery	14 (18.4)	7 (7.2)	0.025	−11.20 (−22.01 – −1.27)
	Structural heart defect^+^	8 (10.5)	7 (7.2)	0.443	−3.31 (−12.93 – 5.30)
	Other arrhythmia	3 (3.9)	6 (6.2)	0.733	2.24 (−5.53 – 9.38)
Medication	Anticoagulation therapy	65 (85.5)	26 (26.8)	<0.001	−58.72 (−68.69 – −45.15)
	Beta-blocker	54 (71.1)	40 (41.2)	<0.001	−29.82 (−42.73 – −14.98)
	Digoxin	12 (15.8)	2 (2.1)	0.001	−13.73 (−23.65 – −5.42)
	Other anti-arrhythmia medication	5 (6.6)	1 (1.0)	0.088	−5.55 (−13.51 – 0.36)
Symptoms prior to hospital admission	Fatigue	47 (61.8)	51 (52.6)	0.222	−9.26 (−23.37 – 5.55)
	General state decline	43 (56.6)	53 (54.6)	0.799	−1.94 (−16.41 – 12.77)
	Respiratory distress	38 (50.0)	25 (25.8)	<0.001	−24.23 (−37.61 – −9.71)
	Heart palpitation	32 (42.1)	21 (21.6)	0.004	−20.46 (−33.69 – −6.53)
	Chest pain	17 (22.4)	18 (18.6)	0.573	−3.81 (−16.21 – 8.08)
	Dizziness or syncope	5 (6.6)	1 (1.0)	0.088	−5.55 (−13.51 – 0.36)
	Fever	3 (3.9)	2 (2.1)	0.655	−1.89 (−9.07 – 3.88)
	Abdominal pain	2 (2.6)	2 (2.1)	1.000	−0.57 (−7.21 – 4.92)
	Numbness, tingling or paralysis	1 (1.3)	2 (2.1)	1.000	0.75 (−5.21 – 6.01)
	Nausea	1 (1.3)	1 (1.0)	1.000	−0.28 (−6.11 – 4.42)
	Headache	0 (0.0)	4 (4.1)	0.132	4.12 (−1.30 – 10.13)
	Back pain	0 (0.0)	4 (4.1)	0.132	4.12 (−1.30 – 10.13)
	Other symptoms	7 (9.2)	11 (11.3)	0.649	2.13 (−7.76 – 11.25)
Symptoms during the registration^#^	Fatigue	28 (59.6)	21 (41.2)	0.069	−18.40 (−36.22 – 1.33)
	General state decline	14 (29.8)	14 (27.5)	0.798	−2.34 (−19.89 – 15.16)
	Respiratory distress	17 (36.2)	13 (25.5)	0.252	−10.68 (−28.09 – 7.43)
	Heart palpitation	13 (27.7)	8 (15.7)	0.149	−11.97 (−27.95 – 4.36)
	Chest pain	4 (8.5)	6 (11.8)	0.743	3.25 (−9.77 – 15.96)
	Dizziness or syncope	0 (0.0)	1 (2.0)	1.000	1.96 (−5.77 – 10.30)
	Fever	0 (0.0)	0 (0.0)	NA	NA
	Abdominal pain	1 (2.1)	0 (0.0)	0.480	−2.12 (−11.11 – 5.09)
	Numbness, tingling or paralysis	1 (2.1)	0 (0.0)	0.480	−2.12 (−11.11 – 5.09)
	Nausea	0 (0.0)	0 (0.0)	NA	NA
	Headache	0 (0.0)	1 (2.0)	1.000	1.96 (−5.77 – 10.30)
	Back pain	0 (0.0)	1 (2.0)	1.000	1.96 (−5.77 – 10.30)
	Other symptoms	0 (0.0)	1 (2.0)	1.000	1.96 (−5.77 – 10.30)

* Mean difference and (95% confidence interval of the difference) values for Age, BMI, HR, and recording duration are years, kg/m^2^, 1/min, and hours.

+ Including valvular defects and congenital defects.

# AF n=47, SR n=51.

Values are mean ± standard deviation and number (percentages). In the last column, values are mean difference (95% confidence interval of the difference lower and upper bound). Values for dichotomical variables in this column (e.g., sex or hypertension) are percentages. Other symptoms included sweating, pains, swelling, sleep, and urinating problems. AF, atrial fibrillation; CI, confidence interval; kg, kilogram; m, meter; min, minute; NA, not applicable; and SR, sinus rhythm.

### Duration of Recordings and Heart Rate

The mean duration of the recordings was 22.0 ± 3.0 h in the AF group and 21.8 ± 3.4 h in the SR group (*p* = 0.705). In the AF group, 39.5% of the recordings and in the SR group 40.2% of patients reached the targeted duration of 24 h (*p* = 0.923). Recordings were discontinued before reaching the targeted duration due to patient or treatment-related reasons or if the patient was discharged from the hospital.

Mean heart rate was higher in the AF group than in the SR group (78.0 ± 18.8 bpm vs. 66.5 ± 11.8 bpm, *p* < 0.001).

### Quality of PPG Data for AF Screening

**Figure 3:**
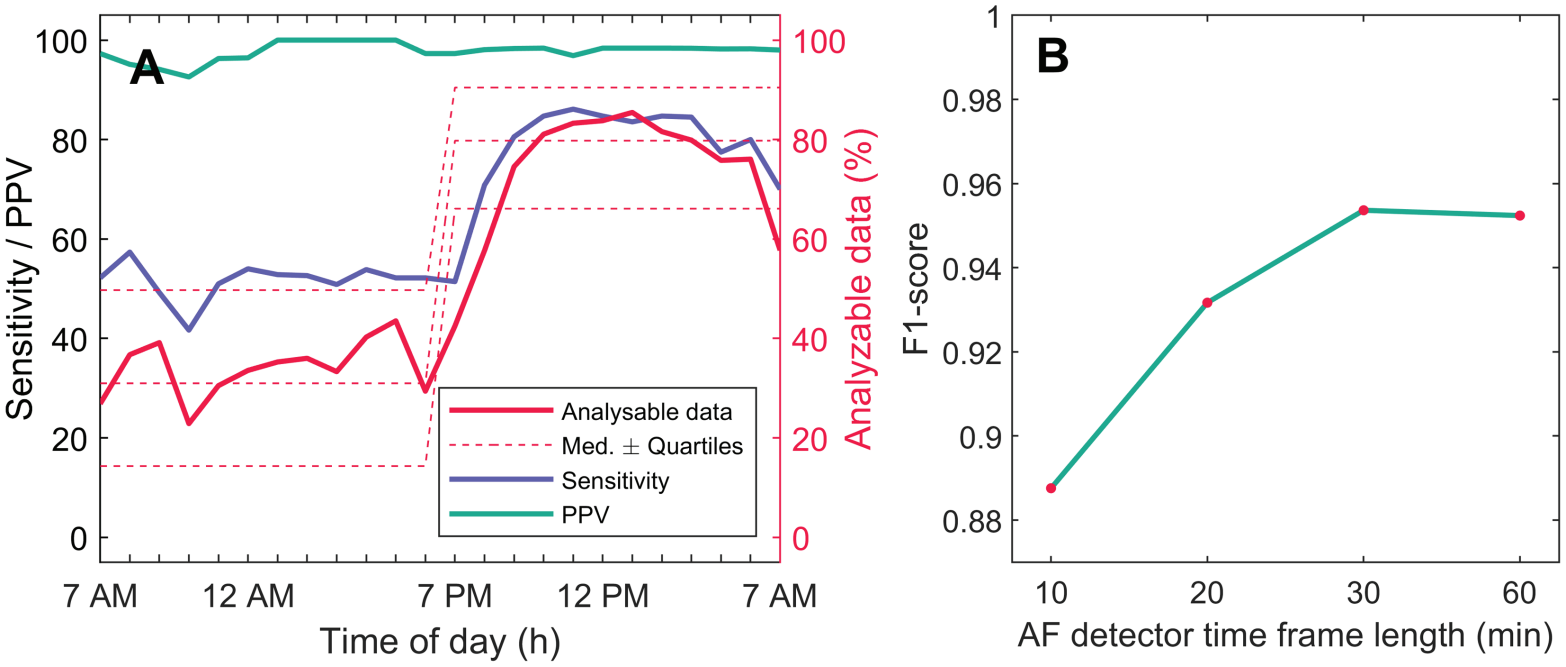
AF detection performance and PPQ quality. **(A)** shows AF detection mean sensitivity (blue) and PPV (green) calculated for each hour of day. Simultaneous 3-lead ambulatory ECG recording served as rhythm control. Hourly analyzable PPG data amount is shown with solid red line, and median quartiles with dashed red line. **(B)** shows F1-scores for patient-based AF detection for AF detection time-frames of 10, 20, 30, and 60 min. AF, atrial fibrillation; AM, *ante meridiem*; h, hour; Med, median; min, minute; PM, post meridiem; and PPV, positive predictive value.

A total of 3781 h of PPG data were analyzed, and 2114 h (55.9%) of the data were approved by the quality algorithm. The quality of PPG data was higher at night (7 PM–7 AM) than during the daytime [7 AM–7 PM; accepted data 67.3 ± 22.4% vs. 30.5 ± 19.4%; mean difference − 36.79 (95% confidence interval, CI, lower −34.00 to upper bound −39.59), *p* < 0.001; Figure 3A].

The mean total duration of analyzable PPG data was 52.5 ± 23.4% in the AF group and 59.5 ± 20.0% in the SR group [−6.96 (−13.49–−0.43), *p* = 0.037]. The proportion of PPG data that was rejected by the quality algorithm based on acceleration data (hand-movement) did not differ between the groups: AF group 30.0 ± 12.6% vs. SR group 28.9 ± 13.2% [10.57 (−2.89–4.97), *p* = 0.595]. However, PPG data rejection based on PPG stationarity was higher in the AF group: AF group 17.5 ± 15.3% vs. SR group 11.6 ± 12.2% [5.90 (1.66–10.15), *p* = 0.007].

### AF Detection and Simulated Alarms

Time-based and patient-based AF detection sensitivities and specificities for PPG data approved by the quality algorithm are presented in Table 2. The time-based AF detection sensitivities were 94.5–95.0% and specificities 98.2–98.7% depending on which detector time-frame was applied. Correspondingly, patient-based AF detection sensitivities were 92.1–98.7% and specificities 81.4–99.0%. The F1-scores for patient-based AF detection as a function of AF detector time-frame length of 10, 20, 30, and 60 min are presented in Figure 3B. The 30-min AF detector time-frame yielded the highest F1 score of 0.9536. When using the 30-min AF detection time, 72/76 AF patients were correctly identified (sensitivity 94.7%) and only 3/97 SR patients received a false positive AF diagnosis (specificity 96.9%).

**Table 2:** Sensitivities and specificities of AF detection from different AF detection time-frames.

		10 min	20 min	30 min	60 min
Patient-based	Sensitivity	98.6	98.7	94.7	92.1
	Specificity	81.4	89.7	96.9	99.0
Time-based	Sensitivity	95.0	95.0	94.9	94.5
	Specificity	98.2	98.4	98.6	98.7

Values were calculated for each patient and for all combined PPG data (time-based). Patient sensitivity was calculated with the condition if the AF detection algorithm diagnosed a patient with at least one true AF and specificity if a patient with SR was diagnosed with even one AF diagnosis by the algorithm from the whole PPG registration. For the time-based results, every detector window diagnosed as AF by the algorithm was a true AF if that same window was diagnosed as AF in the 3-lead ECG interpreted by an investigator.

The 30-min AF detector time-frame yielded correct AF alarms with mean hourly sensitivity of 66.0% and a PPV of 97.8% (Table 3). During the night-time, the hourly sensitivity and PPV were 78.2 and 97.2%; during the daytime, the corresponding values were 49.3 and 97.0%.

**Table 3:** Performance of simulated AF alarms.

		10 min	20 min	30 min	60 min
AF alarms (AF group patients, *n* = 76)	Sensitivity	73.8%	69.1%	66.0%	62.1%
	PPV	94.7%	96.6%	97.8%	98.1%
False AF alarms (SR group patients /patients with no AF, *n* = 97)	Patients	18 (18.6%)	10 (10.3%)	3 (3.1%)	1 (1%)
	Average per patient	3.5	3.8	7.7	19
False AF alarms (AF group patients with paroxysmal AF and SR, *n* = 11)	Patients	2 (18%)	0	0	0
	Average per patient	1	0	0	0

Simulated AF alarms were generated for each hour over the entire study population to simulate the continuous use of the AF detection PPG wrist-band device. All data were divided into 1-h segments and sensitivity was calculated as correct hours with AF alarms/h with true ECG-confirmed AF. AF, atrial fibrillation; PPV, positive predictive value; and SR, sinus rhythm.

No false AF alarms were given to patients with both paroxysmal AF and SR in their recordings with time-frames of 20, 30, and 60 min, but two paroxysmal AF patients received a false AF alarm during SR with the 10-min time-frame. With the 30-min AF time-frame, nine of the eleven patients with paroxysmal AF had 100% of their AF episodes and one patient 35.0% correctly diagnosed with AF, and one paroxysmal AF patient was left without an AF alarm.

### User Experience

A total of 135 (78.0%) patients filled in the user experience survey. The patients graded the PPG wrist-band as more comfortable than the Holter ECG device (2.50 ± 0.91 vs. 2.72 ± 0.82), respectively [0.22 (95% CI 0.08–0.37), *p* = 0.003]. Altogether 82.2% of the patients would be willing to use the wrist-band at home for rhythm monitoring. Among the patients aged ≥65 years, 77.6% responded that they would use the wrist-band at home.

If we assess the comments made about the wrist-band, only 6 (4.4%) of the patients felt that it interfered with their eating, sleeping, and/or going to the toilet. Only 2 (1.5%) of the patients reported that the wrist-band interfered with their ability to move. More of the respondents (14 = 10.4%) stated that the Holter device interfered with eating, sleeping, and/or going to the toilet. The Holter device interfered with sleeping more often than the wrist-band [4 vs. 11; −0.06 (−0.11–−0.01); *p* = 0.011].

Three (2.2%) of the patients reported sweating of the wrist during the PPG recording. Two (1.5%) of the patients experienced adverse effects from the Holter wet electrode glue, including itching and irritation of skin; in two other cases (1.5%), there were problems with the electrodes falling off.

## Discussion

Our study demonstrated that continuous wrist-band PPG monitoring with an automated algorithm detected AF with high sensitivity and specificity from selected good quality samples. According to recent guidelines, PPG alone is not sufficient for AF diagnosis (Hindricks et al., 2021). However, PPG-based AF alarms followed by timely ECG recording for rhythm confirmation could be useful for AF screening. In addition, the patients graded PPG as more comfortable than Holter recording; the majority of the patients was willing to use a PPG wrist-band at home for rhythm monitoring. The study was conducted in a clinical setting rather than using database data and 200 patients were prospectively recruited. Currently, the scientific data concerning AF detection with continuous PPG monitoring in ambulatory patients is limited, and there is lack of guidelines for PPG-based AF screening. Our study suggests that a wearable PPG is useful for long-term screening of asymptomatic and paroxysmal AF.

Photoplethysmography has been previously proven to be able to detect AF (Bashar et al., 2019; Dörr et al., 2019; Fan et al., 2019; Kashiwa et al., 2019; Sološenko et al., 2019; Väliaho et al., 2019; Avram et al., 2021; Väliaho et al., 2021), but the signal quality is affected by motion artifacts (Bashar et al., 2019; Sološenko et al., 2019; Nabavi et al., 2020). Recently, in a continuous AF monitoring study a total of 32.2% of the PPG data was classified as indeterminate, and in line with us more often during the day-time compared to night-time (Avram et al., 2021). In our study, during the daytime, as much as 69.5% of the data recorded from ambulatory patients were discarded. This demonstrates that hand-movement causes significant motion artifacts in the PPG signal. In previous studies in stationary patients at rest, 5.5–43.2% of the data from short 1–5 min PPG recordings also were discarded due to insufficient signal quality (Dörr et al., 2019; Fan et al., 2019). In a study evaluating PPG wrist-band signal quality in mobile patients with SR, up to 87.2% of 30-s PPG segments were discarded due to both motion and noise artifacts, and 68.0% when only motion artifacts (acceleration) were considered (Bashar et al., 2019). In line with these reports, here 32.7% of the night-time data recorded from resting patients were deemed non-interpretable; of this, almost one-third could not be explained fully by hand-movement, and 69.5% of the daytime data were discarded. As the PPG is recorded with an optical sensor, proper skin contact is needed for good quality recording, i.e., the position of the sensor can affect the signal quality even when the hand is stationary. In the future development of sensor fusion algorithms compensating for signal disturbances caused by acceleration and sensor position will be needed to improve PPG signal quality, particularly during the daytime (Nabavi et al., 2020). In our study, the proportion of rejected PPG data based on acceleration was similar between the groups, but more data were discarded in the AF group compared to the SR group based on PPG waveform stationarity. However, in the validation process, there was no difference in detection of high-quality 1-min segments between AF and SR groups. The decreased PPG waveform stationarity in the AF group is probably related to the higher heart rate in AF, which may alter beat-to-beat stroke volumes and thereby distort PPG waveform amplitudes causing merging of some pulse waves.

PPG-based AF detection in stationary patients from short, a few minute long recordings, can provide AF detection with high sensitivity (93.7-96.4%) and specificity (96.3-99.7%) (Dörr et al., 2019; Fan et al., 2019; Väliaho et al., 2019; Väliaho et al., 2021). With continuous PPG monitoring, sensitivities of 72.0–92.7% and specificities of 97.4–99.7% have been reported (Bashar et al., 2019; Kashiwa et al., 2019; Sološenko et al., 2019; Avram et al., 2021) and discarding poor quality data decreased portion of false positive AF diagnoses (Bashar et al., 2019; Sološenko et al., 2019). Despite the problem of a non-interpretable signal and 44.1% of total discarded PPG data, the current study displayed a sensitivity of 95.4%, a specificity of 99.7% and PPV of 97.8% for the detection of AF in a clinical setting in ambulatory patients and 24-h continuous PPG recordings. Only 3 patients here received false AF alarms, as low-quality PPG samples were discarded. Our findings also confirmed some earlier results (Avram et al., 2021), that AF can be diagnosed more likely during the night compared to day-time. In resting patients with continuous PPG monitoring, AF was identified with a sensitivity of 81.0%, specificity of 96.4%, and PPV of 86.6% (Kashiwa et al., 2019). In our study from night-time recordings, the hourly simulated AF alarm sensitivity for the detection of AF was similar 78.2%, but the PPV was higher 97.2%. We also found that during daytime, the sensitivity of hourly AF alarm declined significantly, which is logical as more data are rejected due to poor PPG quality caused by the hand-movement. For example, if a 30-min PPG segment during AF rhythm was interpreted to contain too much low-quality data and thus not qualified for rhythm analysis, no AF alarm was given and this was counted as false negative AF detection, which decreased AF detection sensitivity in our results. Nonetheless, our method provided reliable AF detection alarms with high PPV during continuous PPG monitoring at any time (97.0–97.2%). The AF detection sensitivity was higher at night when the patient was resting. As PPG is susceptible to artifacts caused by noise or motion which can lead to false AF alarms, a higher specificity for detection AF is likely to be preferred in exchange for a lower sensitivity in continuous PPG screening lasting at least for a day (Sološenko et al., 2019). False alarms during continuous PPG monitoring could become an unwieldy problem, that could restrict feasibility of the approach of PPG-based screening followed by an ECG for rhythm verification (Sološenko et al., 2019). We demonstrated a continuous monitoring method with high PPV for AF achieved with PPG signal quality assessment and relatively long duration detection time-frames. Currently, the state-of-the-art PPG-based AF detectors can identify AF episodes with high accuracy from high-quality data. In the future, a special focus should be on developing sensor mechanics and methods for compensating for the motion artifacts to improve PPG quality and AF detection accuracy.

The special demands of the elderly, the main target population of AF screening, are not usually given sufficient consideration in the development of wearable health technologies (Ehmen et al., 2012). Wrist-band activity trackers have been found to be easy to use by the elderly in their daily lives and to generally improve health outcomes in this population (O’Brien et al., 2015). In a feasibility study of wrist-band activity trackers among the elderly, the participants wore these devices 24 h a day for 12 weeks with only 15% of them terminating wearing the wrist-bands (O’Brien et al., 2015). In our study, the majority of the patients (82.2%) reported that they would be willing to use the PPG wrist-band at home for rhythm monitoring. O’Brien et al. reported that the patients in their study mastered the use of an activity wrist-band easily for sport and health purposes (O’Brien et al., 2015). Thus, it does seem that “around-the-clock” use of the wrist-band is well tolerated.

There are increasing data supporting the proposal that the risk of stroke increases with AF burden, but the current guidelines regard AF as either absent or present (Tiver et al., 2021). The effect of brief AF episodes (lasting up to 6 min) on the risk of stroke remains unknown (Chen et al., 2018). Brief AF episodes or atrial high rate episodes lasting from 5 or 6 min to 1 h have been associated with a 2-fold risk of ischaemic stroke (Healey et al., 2012; Boriani et al., 2014), but during 2-year follow-up, AF burden of 1 h was found to be associated with the highest hazard ratio for ischaemic stroke (vs. 5-min, 6-h, 12-h, and 23-h daily AF burden; Boriani et al., 2014). We tested the performance of AF detector frames of 10- to 60-min duration. We found that a shorter AF detector frame yielded higher sensitivity but a longer window yielded higher specificity. The 30-min frame achieved the best performance for AF detection.

AF can be silent, and it is a major preventable risk factor for stroke. Screening of AF is justified among the high-risk groups, including the elderly and/or patients with other risk factors for stroke. The diagnostic yield for AF increases with the duration, dispersion and number of the screenings (Tung et al., 2015; Chua et al., 2020; Diederichsen et al., 2020). In the long-term continuous monitoring of patients with stroke and transient ischaemic attacks, the first episode of paroxysmal AF was usually detected after 1.5 days of monitoring (Tung et al., 2015). Small ECG devices designed for long-term rhythm monitoring, such as ECG patches, have been proposed to be beneficial for AF screening. In a study with 14-day continuous ECG monitoring, 7-fold more new AF or supraventricular tachyarrhythmia cases were detected compared to standard 24-h ambulatory ECG monitoring in patients with a mean age of 62 years (Chua et al., 2020) and 10-fold more AF cases as compared to standard care with regular pulse checks and heart auscultations in patients aged 75 or older (Gladstone et al., 2021). However, ECG monitoring with ambulatory ECGs or single ECG patches requires application of wet electrodes, which can cause skin irritation and decrease the device’s comfort, particularly during long-term recording Gladstone et al. reported 1.2% of the patients reported skin irritation caused by the ECG patches (Gladstone et al., 2021) but Chua et al. reported a much higher value (46%) (Chua et al., 2020). Here, only 1.5% patients complained of skin reactions from the wet electrodes of the ambulatory ECG used as the golden standard for rhythm analysis. A PPG wrist-band does not need wet electrodes or electrical wires, and the device can be easily removed and re-worn by the user. Notably, the wrist-band did not evoke any skin reactions. Thus, we would hypothesize that in terms of user-friendliness, PPG is more feasible for long-term rhythm screening than ECG recording.

### Limitations

The study was conducted in a hospital environment, which influenced the extent to which the patients engaged in physical activity. Although after the first 5 min, the patients were allowed to move freely in the hospital, the patients were likely to follow the time schedules in the hospital wards, e.g., same dinner-times and roughly same sleep rhythm. The performance of PPG recording for AF detection needs to be confirmed using ambulatory recordings outside a hospital setting.

Although the study data were collected prospectively, the sampling and analyzing were undertaken retrospectively with the extracted PPG signal data. Rhythms were only classified as AF or SR by the algorithm and no other arrhythmias except if extrasystoles were present in the recordings. Only 14.5% of the AF patients had paroxysmal AF. The ultimate goal in real-life is to detect asymptomatic and paroxysmal AF in high-risk patients. Here, patients with initially known rhythm (SR or AF) based on the 12-lead ECG were recruited. Thus, the study should be seen as a proof-of-concept, and more research will be needed to evaluate the diagnostic yield of AF in patients with asymptomatic and paroxysmal AF.

The 30-min time-frame was used here for generating AF alarms. This time-frame can only detect AF episodes lasting 30 min, and therefore, shorter AF paroxysms would be left without an AF alarm. However, there are two issues that need to be addressed here. First, a short AF episode confirms that the patients have AFs. Indeed, in spite of the limitations listed above, in our study, nine of the eleven patients with paroxysmal AF (14.5%) had all their AF episodes correctly diagnosed, and only in one patient, all AF episodes were missed. Secondly, recent studies have found that AF episodes lasting only few minutes are not long enough to confirm that the patient has risk of stroke and are not indicative to start anticoagulation (Van Gelder et al., 2017; Svendsen et al., 2021).This suggests that longer AF episodes should be screened to identify patients who benefit from anticoagulation.

The final diagnosis of AF cannot be made based only on PPG. This requires a 12-lead resting ECG or 30-s 1-lead ECG recording (Hindricks et al., 2021). However, long-term PPG screening with AF alarms could trigger a timely ECG recording for the confirmation of AF.

## Conclusion

We demonstrated that 24-h wrist-band PPG monitoring with an automated algorithm provided reliable AF detection. Due to movement artifacts, the quality of the PPG signal was lower during the daytime. Nonetheless, the PPV of AF detection remained high (97.0–97.2%) throughout the 24 h. The patients graded PPG as more comfortable than Holter recording and most of them were willing to use a PPG wrist-band at home for AF monitoring.

## Data Availability Statement

The raw data supporting the conclusions of this article will be made available by the authors, without undue reservation.

## Ethics Statement

The studies involving human participants were reviewed and approved by the Ethics Committee of the Northern Savo Hospital District (approval 347/2018). The patients/participants provided their written informed consent to participate in this study. The participants were all volunteer adult patients. They were given written information about the study.

## Author Contributions

E-SV, JL, PK, TM, HJ, TR, CM, JH, MT, TL, TL, OS, and JH contributed to the design of the study. E-SV and OS contributed to the collection of data. E-SV, JL, and OS performed the data analysis. E-SV and JL performed the statistical analysis. E-SV drafted the manuscript. All authors contributed to the article and approved the submitted version.

## Funding

This work was supported by the Research Committee of the Kuopio University Hospital Catchment Area for the State Research Funding (project 5101137, Kuopio, Finland), the Finnish Medical Foundation, the Ida Montin Foundation, the Finnish Cultural Foundation (North-Savo Regional Fund, A. A. Laaksonen Fund), the Antti and Tyyne Soininen Foundation, the Paavo Nurmi Foundation, and the Finnish Foundation for Cardiovascular Research.

## Conflict of Interest

JL, TR, TM, HJ, JH, and MT are shareholders of a company (Heart2Save) that designs ECG-based software for medical equipment. TM, PK, JL, MT, and HJ report personal fees from Heart2Save.

## Publisher’s Note

All claims expressed in this article are solely those of the authors and do not necessarily represent those of their affiliated organizations, or those of the publisher, the editors and the reviewers. Any product that may be evaluated in this article, or claim that may be made by its manufacturer, is not guaranteed or endorsed by the publisher.

## References

[ref1] Avram R. Ramsis M. Cristal A. D. Nathan V. Zhu L. Kim J.. (2021). Validation of an algorithm for continuous monitoring of atrial fibrillation using a consumer smartwatch. Heart Rhythm. 18, 1482–1490. doi: 10.1016/j.hrthm.2021.03.044, PMID: 33838317

[ref2] Bashar S. K. Han D. Ding E. Whitcomb C. McManus D. Chon K. H. (2019). “Smartwatch based atrial fibrillation detection from Photoplethysmography signals,” in *Proceedings of the Annual International Conference of the IEEE Engineering in Medicine and Biology Society, EMBS*, July 1, 2019. 10.1109/EMBC.2019.8856928 31946820

[ref3] Boriani G. Glotzer T. V. Santini M. West T. M. De Melis M. Sepsi M.. (2014). Device-detected atrial fibrillation and risk for stroke: An analysis of >10,000 patients from the SOS AF project (stroke prevention strategies based on atrial fibrillation information from implanted devices). Eur. Heart J. 35, 508–516. doi: 10.1093/eurheartj/eht491, PMID: 24334432 PMC3930873

[ref4] Chen L. Y. Chung M. K. Allen L. A. Ezekowitz M. Furie K. L. McCabe P.. (2018). American Heart Association Council on Clinical Cardiology; Council on Cardiovascular and Stroke Nursing; Council on Quality of Care and Outcomes Research; and Stroke Council. Atrial Fibrillation Burden: Moving Beyond Atrial Fibrillation as a Binary Entity: A Scientific Statement From the American Heart Association. Circulation 137, e623–e644. doi: 10.1161/CIR.0000000000000568, PMID: 29661944 PMC8463258

[ref5] Chua S. K. Chen L. C. Lien L. M. Lo H. M. Liao Z. Y. Chao S. P.. (2020). Comparison of arrhythmia detection by 24-hour Holter and 14-day continuous electrocardiography patch monitoring. Acta Cardiol. Sin. 36, 251–259. doi: 10.6515/ACS.202005_36(3).20190903A, PMID: 32425440 PMC7220965

[ref6] Diederichsen S. Z. Haugan K. J. Kronborg C. Graff C. Højberg S. Køber L.. (2020). Comprehensive evaluation of rhythm monitoring strategies in screening for atrial fibrillation: insights From patients at risk monitored long term with an implantable loop recorder. Circulation 141, 1510–1522. doi: 10.1161/CIRCULATIONAHA.119.044407, PMID: 32114796

[ref7] Dörr M. Nohturfft V. Brasier N. Bosshard E. Djurdjevic A. Gross S.. (2019). The WATCH AF trial: SmartWATCHes for detection of atrial fibrillation. JACC Clin. Electrophysiol. 5, 199–208. doi: 10.1016/j.jacep.2018.10.006, PMID: 30784691

[ref8] Ehmen H. Haesner M. Steinke I. Dorn M. Gövercin M. Steinhagen-Thiessen E. (2012). Comparison of four different mobile devices for measuring heart rate and ECG with respect to aspects of usability and acceptance by older people. Appl. Ergon. 43, 582–587. doi: 10.1016/j.apergo.2011.09.003, PMID: 21962327

[ref9] Fan Y. Y. Li Y. G. Li J. Cheng W. K. Shan Z. L. Wang Y. T.. (2019). Diagnostic performance of a smart device with photoplethysmography technology for atrial fibrillation detection: pilot study (pre-mAFA II registry). JMIR Mhealth Uhealth 7:e11437. doi: 10.2196/11437, PMID: 30835243 PMC6423467

[ref10] Gladstone D. J. Wachter R. Schmalstieg-Bahr K. Quinn R. Hummers E. Ivers N.. (2021). Screening for atrial fibrillation in the older population: A randomized clinical trial. JAMA Cardiol. 6, 558–567. doi: 10.1001/jamacardio.2021.0038, PMID: 33625468 PMC7905702

[ref11] Hariri E. Hachem A. Sarkis G. Nasr S. (2016). Optimal duration of monitoring for atrial fibrillation in cryptogenic stroke: A nonsystematic review. Biomed. Res. Int. 2016:5704963. doi: 10.1155/2016/5704963, PMID: 27314027 PMC4903126

[ref12] Healey J. S. Connolly S. J. Gold M. R. Israel C. W. Van Gelder I. C. Capucci A.. (2012). ASSERT Investigators. Subclinical atrial fibrillation and the risk of stroke. N Engl. J. Med. 366, 120–129. doi: 10.1056/NEJMoa1105575, PMID: 22236222

[ref13] Hindricks G. Potpara T. Dagres N. Arbelo E. Bax J. J. Blomström-Lundqvist C.. (2021). 2020 ESC guidelines for the diagnosis and management of atrial fibrillation developed in collaboration with the European Association for Cardio-Thoracic Surgery (EACTS): The task force for the diagnosis and management of atrial fibrillation of the European Society of Cardiology (ESC) developed with the special contribution of the European heart rhythm association (EHRA) of the ESC. Eur. Heart J. 42, 373–498. doi: 10.1093/eurheartj/ehaa612, PMID: 32860505

[ref14] Kashiwa A. Koyama F. Miyamoto K. Kamakura T. Wada M. Yamagata K.. (2019). Performance of an atrial fibrillation detection algorithm using continuous pulse wave monitoring. Ann. Noninvasive Electrocardiol. 24:e12615. doi: 10.1111/anec.12615, PMID: 30387545 PMC6931792

[ref15] Morillo C. A. Banerjee A. Perel P. Wood D. Jouven X. (2017). Atrial fibrillation: the current epidemic. J. Geriatr. Cardiol. 14, 195–203. doi: 10.11909/j.issn.1671-5411.2017.03.011, PMID: 28592963 PMC5460066

[ref16] Nabavi S. Bhadra S. (2020). A robust fusion method for motion artifacts reduction in Photoplethysmography signal. IEEE Trans. Instrum. Meas. 69, 9599–9608. doi: 10.1109/TIM.2020.3006636

[ref17] O’Brien T. Troutman-Jordan M. Hathaway D. Armstrong S. Moore M. (2015). Acceptability of wristband activity trackers among community dwelling older adults. Geriatr. Nurs. 36, S21–S25. doi: 10.1016/j.gerinurse.2015.02.019, PMID: 25771957

[ref18] Sološenko A. Petrenas A. Paliakaite B. Sörnmo L. Marozas V. (2019). Detection of atrial fibrillation using a wrist-worn device. Physiol. Meas. 40:025003. doi: 10.1088/1361-6579/ab029c 30695758

[ref19] Svendsen J. H. Diederichsen S. Z. Højberg S. Krieger D. W. Graff C. Kronborg C.. (2021). Implantable loop recorder detection of atrial fibrillation to prevent stroke (The LOOP study): a randomised controlled trial. Lancet 398, 1507–1516. doi: 10.1016/S0140-6736(21)01698-6, PMID: 34469766

[ref20] Tiver K. D. Quah J. Lahiri A. Ganesan A. N. McGavigan A. D. (2021). Atrial fibrillation burden: an update-the need for a CHA2DS2-VASc-AFBurden score. Europace 23, 665–673. doi: 10.1093/europace/euaa287, PMID: 33351904

[ref21] Tung C. E. Su D. Turakhia M. P. Lansberg M. G. (2015). Diagnostic yield of extended cardiac patch monitoring in patients with stroke or TIA. Front. Neurol. 5:266. doi: 10.3389/fneur.2014.00266, PMID: 25628595 PMC4290477

[ref22] Väliaho E.-S. Kuoppa P. Lipponen J. A. Hartikainen J. E. K. Jäntti H. Rissanen T. T.. (2021). Wrist band photoplethysmography autocorrelation analysis enables detection of atrial fibrillation without pulse detection. Front. Physiol. 12:576. doi: 10.3389/fphys.2021.654555, PMID: 34025448 PMC8138449

[ref23] Väliaho E.-S. Kuoppa P. Lipponen J. A. Martikainen T. J. Jäntti H. Rissanen T. T.. (2019). Wrist band photoplethysmography in detection of individual pulses in atrial fibrillation and algorithm-based detection of atrial fibrillation. Europace 21, 1031–1038. doi: 10.1093/europace/euz060, PMID: 31505594

[ref24] Van Gelder I. C. Healey J. S. Crijns H. J. G. M. Wang J. Hohnloser S. H. Gold M. R.. (2017). Duration of device-detected subclinical atrial fibrillation and occurrence of stroke in ASSERT. Eur. Heart J. 38, 1339–1344. doi: 10.1093/eurheartj/ehx042, PMID: 28329139

