# Supplementary material for: Continuous 24-h Photoplethysmogram Monitoring Enables Detection of Atrial Fibrillation
Source: Front Physiol. 2022 Jan 4;12:778775. doi: 10.3389/fphys.2021.778775 (PMC8764282; doi:10.3389/fphys.2021.778775)
Supplement: Supplementary file 2 [file Data_Sheet_2.PDF]

Supplementary Appendix 2 – PPG quality algorithm validation

Performance of PPG quality algorithm was evaluated with subpopulation of 50 subjects (AF N = 25, SR N = 25). ECG and PPG measurements of randomly selected patients where synchronized. Positive predictive value (PPV) of pulse detection were evaluated for all 1-minute segments, by using method presented in ANSI/AAMI EC57: 2012 clause 4.3.[1] 1-minute segments with >95% pulse detection PPV were deemed as high-quality samples and PPV ≤ 95 as low-quality samples, which were used as a “correct” reference values in assessing performance of PPG quality algorithm. Performance measures of PPG quality algorithm are presented in Supplementary Appendix 2 Table 1.

Supplementary Appendix 2 Table 1. Performance measures of PPG quality algorithm

	Number of samples	Sensitivity (%)	PPV (%)	Specificity (%)	NPV (%)
All samples	61679	89.0	92.7	91.4	90.8
SR	30945	89.7	91.4	92.4	90.9
AF	30734	88.3	91.4	93.2	90.7

Abbreviations: AF, atrial fibrillation; PPV, positive predictive value; NPV, negative predictive value.

Supplementary Appendix 2 References

1. American National Standards Institute (2012). Testing And Reporting Performance Results Of Cardiac Rhythm And ST Segment Measurement Algorithms (ANSI/AAMI EC57:2012).
