# Supplementary figures and images for: Continuous 24-h Photoplethysmogram Monitoring Enables Detection of Atrial Fibrillation
Source: Front Physiol. 2022 Jan 4;12:778775. doi: 10.3389/fphys.2021.778775 (PMC8764282; doi:10.3389/fphys.2021.778775)

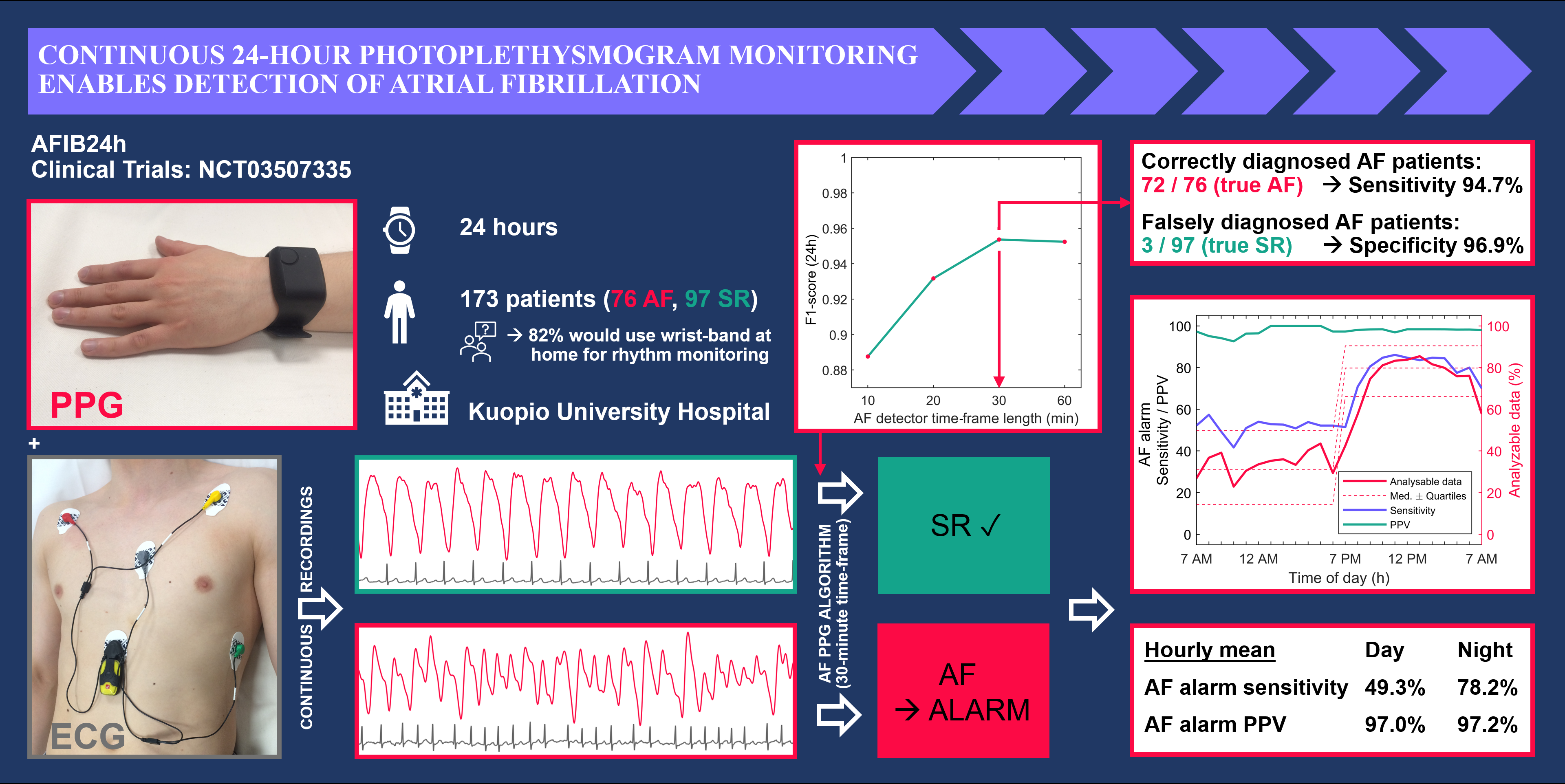

Supplement: Supplementary file 3 [file Image_1.TIF]
